# Life-history responses to changing temperature and salinity of the Baltic Sea copepod *Eurytemora affinis*

**DOI:** 10.1007/s00227-017-3279-6

**Published:** 2018-01-18

**Authors:** Konrad Karlsson, Simona Puiac, Monika Winder

**Affiliations:** Department of Ecology, Environment, and Plant Sciences, Stockholm University, 10691 Stockholm, Sweden

## Abstract

To understand the effects of predicted warming and changing salinity of marine ecosystems, it is important to have a good knowledge of species vulnerability and their capacity to adapt to environmental changes. In spring and autumn of 2014, we conducted common garden experiments to investigate how different populations of the copepod *Eurytemora affinis* from the Baltic Sea respond to varying temperatures and salinity conditions. Copepods were collected in the Stockholm archipelago, Bothnian Bay, and Gulf of Riga (latitude, longitude: 58°48.19′, 17°37.52′; 65°10.14′, 23°14.41′; 58°21.67′, 24°30.83′). Using individuals with known family structure, we investigated within population variation of the reaction norm (genotype and salinity interaction) as a means to measure adaptive capacity. Our main finding was that low salinity has a detrimental effect on development time, the additive effects of high temperature and low salinity have a negative effect on survival, and their interaction has a negative effect on hatching success. We observed no variation in survival and development within populations, and all genotypes had similar reaction norms with higher survival and faster development in higher salinities. This suggests that there is no single genotype that performs better in low salinity or high salinity; instead, the best genotype in any given salinity is best in all salinities. Genotypes with fast development time also had higher survival compared to slow developing genotypes at all salinities. Our results suggest that *E. affinis* can tolerate close to freshwater conditions also in high temperatures, but with a significant reduction in fitness.

## Introduction

Climate change is affecting biodiversity of marine organisms, and particularly in coastal estuarine ecosystems like the Baltic Sea that will experience some of the greatest changes in temperature and salinity (Lehmann et al. 2011; Meier et al. 2006). To survive, grow, and reproduce under climate change, organisms have to adapt to the new environmental conditions (Davis and Shaw 2001) or migrate to new habitable areas (Parmesan 2006). Due to taxon-specific tolerance limits, it is difficult to predict how species will cope and interact with environmental changes. A good knowledge of adaptive capacity and tolerance limits of key species can help to improve our understanding of how marine communities will reorganize because of environmental changes. Calanoid copepods are a major trophic link between primary producers and fish (Stibor et al. 2004; Tomczak et al. 2012), and highly important for maintaining fish stocks (Möllmann and Köster 2002). Consequently, it is important to understand how copepods are able to adapt to environmental changes.

Development time and reproduction are two key life-history traits that influence the population dynamics of calanoid copepods (Allan 1976). Both life-history traits are highly variable as a function of environmental conditions. Under optimal growth conditions, most of the available energy is allocated to reproduction after accounting for metabolic costs. Therefore, egg production is often used to estimate copepods optimum temperature (Hirche 1992; Holste and Peck 2006; Huntley and Lopez 1992). An increase in temperature is associated with faster development, a reduction in female’s body size, and smaller egg clutches due to her smaller size (Ban 1994; Blaxter et al. 1998; Gillooly et al. 2001, 2002). Salinity is another important environmental factor affecting the distribution of copepod populations (Devreker et al. 2004; Holste and Peck 2006; Roddie et al. 1984). Under osmotic stress, more energy is allocated for osmoregulation and less for development and egg production, which on a long temporal scale could negatively impact the recruitment potential, and the population rate of increase (Allan 1976). The ability to cope with salinities outside the optimal range is also influenced by temperature (Bradley 1986; Devreker et al. 2009; Lance 1963; Nagaraj 1988), and therefore, interactive effects of these two factors are important for assessing the response of copepod populations to abiotic change.

*Eurytemora affinis* (Poppe) is a euryhaline calanoid copepod with a wide distribution in the Northern hemisphere. It commonly inhabits brackish systems, often being a dominant species in zooplankton communities of European (Escaravage and Soetaert 1995; Gasparini et al. 1999; Peitsch et al. 2000) and North American (Heinle and Flemer 1975; Laprise and Dodson 1994; Winkler et al. 2003) coasts and estuaries. Despite its preference for brackish conditions, some populations of this species can be found in such diverse conditions as freshwater and hypersaline marches (Lee and Petersen 2003); therefore, tolerance limits can vary between populations, and populations demonstrate a high adaptability to salinity as a result of strong osmoregulation (Kimmel and Bradley 2001; Roddie et al. 1984).

*Eurytemora affinis* is an egg-carrying species and after hatching undergoes a nauplius (larval) and a copepodid (juvenile) phase before reaching adulthood (Katona 1971). The larval phase consists of six nauplius stages at the end of which the animals metamorphose into juveniles, comprising five copepodid stages, and further molt into the final adult life stage where sexual reproduction occurs. Feeding (Meunier et al. 2016), mortality (Beyrend-Dur et al. 2009), swimming behavior (Holliland et al. 2012; Schmitt et al. 2011), and predation pressure (van Someren Gréve et al. 2017) all differ between developmental stages. Consequently, the effects of environmental factors can vary between life-history stages.

The goals of the present study were to investigate how life-history traits of different *E. affinis* populations from the Baltic Sea respond to changes in temperature and salinity, and if local origin has a role in their ability to cope with environmental changes. By investigating the response of related individuals to the environment, we aim to estimate the genotype-by-environment interaction as a sign of adaptive capacity (Dam 2013). The Baltic Sea is one of the fastest warming ecosystems, and by the end of the current century, the mean sea surface temperature is predicted to increase by 2–4 °C (Lehmann et al. 2011). In addition, salinity is expected to decrease by 1.5–2 practical salinity units (PSU) because of increasing river run-off (Meier et al. 2006). A good understanding of how *E. affinis* populations respond to environment conditions is highly needed to predict their capability to adapt to future climate scenarios.

## Materials and methods

### Sampling and culture maintenance

**Fig. 1:**
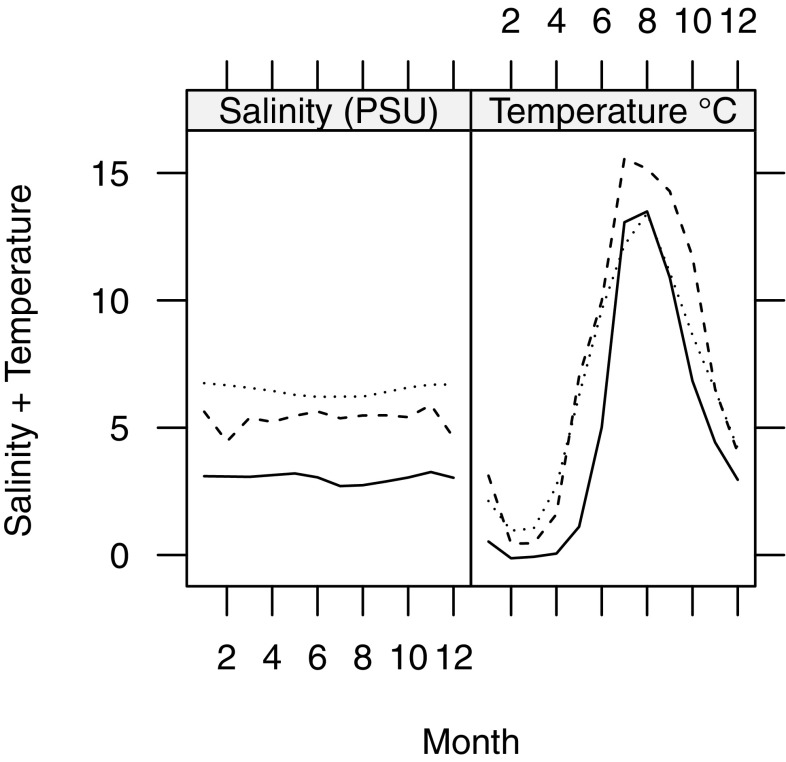
Monthly mean salinity (left panel) and temperature (right panel) at the sampling locations of our test populations at ≤ 30 m depth, the dotted line shows STHLM population, dashed shows GOR, and continuous line shows BB

Copepods were collected from three different areas of the Baltic Sea along the temperature and salinity gradient using a zooplankton net with 90 µm mesh size. A minimum of 300 adult *E. affinis* from each location were sorted out to establish lab cultures. One population was collected in October 2013 from the Stockholm archipelago at the Askö monitoring station B1 (STHLM) (58°48.19′, 17°37.52′); the other two populations were collected in August and September 2014 from Pärnu Bay in the Gulf of Riga (GOR) (58°21.67′, 24°30.83′) and monitoring station F3A5 in the Bothnian Bay (BB) (65°10.14′, 23°14.41′). *E. affinis* mainly inhabit the upper 30 m (Holliland et al. 2012) and monthly mean salinity and temperature at this depth range differed between the locations where the populations were sampled (Fig. 1). Data were available from the Swedish Hydrological and Metrological Institute (SMHI) for station B1 and F3A5, and the International Council for the Exploration of the Seas (ICES) for Pärnu Bay (geographical cutoff: highest lat, lon 58° 35.00′, 24° 47.17′; lowest 58° 02.50′, 24°17.17′). All available observations were used to calculate monthly means of salinity and temperature.

The lab cultures were kept in separate 10 l plastic buckets at 15 °C and salinity of 6 PSU, under constant gentle aeration and a 12 l: 12 D photoperiod. Populations were cultured with artificial seawater, obtained by mixing Instant Ocean^®^ sea salt with tap water. Water was changed twice a week and copepods were fed ad libitum (100,000–200,000 cells ml^−1^) with the cryptophyte algae *Rhodomonas salina*, cultured on an F2 media (Guillard and Ryther 1962) at 10 PSU. All populations were acclimated to the laboratory conditions for at least three generations to avoid imprints of maternal effects (Sanford and Kelly 2011).

### Experimental setup

Two experimental setups were used in the present study. The first one allowed investigating temperature-salinity responses of the STHLM population, while for the GOR and BB, only the effect of salinity was analyzed. Despite the different protocols, this provides general information on the response of *E. affinis* populations to temperature and salinity changes.

For the STHLM population, we used full siblings by pairing C5 female copepodites with one male; all females matured into adults in a few days and mated with the male. This procedure assured that all individuals from each female were full siblings. These pairs were put in 25 ml glass beakers, at either 15 °C and 2 PSU, 15 °C and 6 PSU, 20 °C and 2 PSU, or 20 °C and 6 PSU, we counted the number of eggs per clutch and used eggs (put in 10 ml glass beakers) from these pairs to investigate hatching success. Once the eggs hatched, nauplii were transferred individually to 10 ml glass beakers and development time and survival were recorded until adulthood. For the GOR and BB population, we used maternal siblings by selecting egg-carrying females from the lab cultures; for these two populations, we recorded hatching success, development time, and survival. Each female clutch was crossed over a salinity gradient (0.5, 5, 10 and 15 PSU) at a temperature of 22.5 °C, into two 10 ml vials per salinity treatment. This setup allowed us to investigate if the response to salinity is different between sibling clutches (hereafter siblings is referred to as genotypes). Visual inspections of the animals were performed every day or every other day for the STHLM population, and every day for the GOR and BB populations to assess life stage and survival.

For all populations, development time and survival was assessed on three different life stages according to Katona (1971): from nauplii stage 1 to adult (hereafter adult survival/development), from nauplii stage 1 to copepodid stage 1 (hereafter nauplii survival/development), and from copepodid stage 1 to adult (hereafter copepodid survival/development).

Replication in this study was at the level of genotype, and within each genotype, the number of individuals varied (Table 1). The use of genotype as random variable does two things: first, we avoid pseudo-replication, since individuals with the same parents are not independent. Second, in the GOR and BB population, genotypes were crossed over salinity treatments, which allowed us to quantify variation in genotypes over different salinities, which is a type of interaction between genotype and the environment (Bolker et al. 2009).

**Table 1:** Number of genotypes (random effect) in each treatment combination in the middle columns, and the sum of observations or individuals over all treatment effects in the rightmost column and leftmost column show the separate response variables

Response var.	15 °C and 2 PSU	15 °C and 6 PSU	20 °C and 2 PSU	20 °C and 6 PSU	*n* obs./ind.
*n* genotypes, STHLM					
Clutch size	10	10	13	9	139
Surv. adult	8	5	8	10	356
Surv. cope.	8	5	7	10	219
Dev. adult	7	5	7	10	200
Dev. cope.	7	5	7	10	200
Hatch	9	5	8	8	1692

Response var.	0.5 PSU	5 PSU	10 PSU	15 PSU	*n* ind.
*n* genotypes, GOR & BB					
Surv. adult GOR	9	9	9	9	190
Surv. cope. GOR	9	9	9	9	121
Dev. adult GOR	9	9	9	9	98
Dev. cope. GOR	9	9	9	9	119
Hatch GOR	9	9	9	9	368
Hatch BB	6	6	6	6	178

### Statistical analysis

All analyses were computed in R (R Core Team 2016). We used generalized linear mixed model glmer for the analyses of clutch size, hatching success, survival, and development time (Bates et al. 2015). Clutch size (number of eggs) and development time (days) were analyzed as count data, whereas hatching success and survival were analyzed as proportions, i.e., hatched or not hatched and alive or dead, which is binomial data. Salinity and temperature were treated as fixed factors and genotype was treated as random effect. We also tested the influence of development time on survival; here, development time and salinity were set as fixed continuous effects and genotype as random effect. Due to low survival in the BB population, we excluded statistical tests of survival and development time analysis for this population; however, we included estimates in the figures.

To test for differences in the response of development and survival in different salinity by individual genotypes in the GOR population, we tested two generalized models (glm) against each other: one model with salinity and genotype as fixed effects (genotype + salinity) and one with main effects and their interaction (genotype + salinity + genotype × salinity) in an analysis of deviance.

For clutch size, hatching success, and survival depending on development time, mixed effect model outputs were analyzed as type two ANOVA, using the car package (Fox and Weisberg 2011). In models with non-significant interaction effect, we removed the interaction term and only analyzed main effects according to Engqvist (2005), and therefore, we do not present non-significant interaction terms in the results. Graphical outputs were made with the lattice (Sarkar 2008) and ggplot2 (Wickham 2016) packages; model estimates and confidence intervals for graphs were calculated by the effects package (Fox 2003).

## Results

### Stockholm archipelago population

**Fig. 2:**
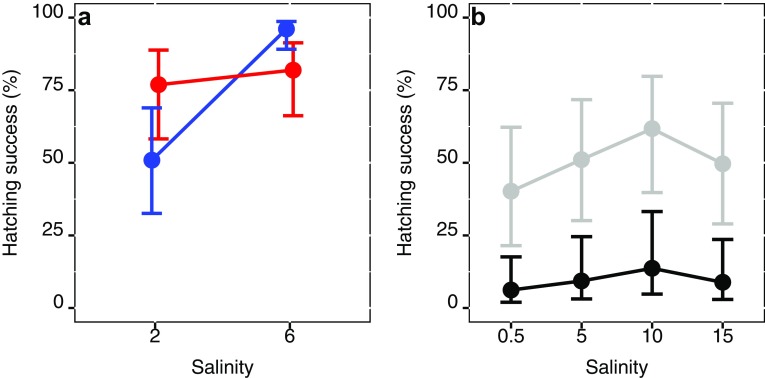
Hatching success in percentage as a response to salinity of **a**, the STHLM population at 15 °C (blue) and 20 °C (red). And **b**, of the GOR (grey) and BB (black) populations. Error bars are confidence limits

We found the number of eggs per clutch to be unaffected by salinity (*df* = 1, *χ*^2^ = 0.24, *p* = 0.68) and temperature (*df* = 1, *χ*^2^ = 1.95, *p* = 0.16), and was on average 20.89 ± 1.03 SE. Salinity and temperature had a significant interactive effect on hatching success (*df* = 1, *χ*^2^ = 9.67, *p* = 0.00188, Fig. 2a), indicating that the sensitivity to salinity differs between temperatures. The highest hatching success (96%) was found at 15 °C and 6 PSU, and the lowest (50%) at 15 °C and 2 PSU. The effect of temperature on hatching success was not significant by itself (*df* = 1, *χ*^2^ = 0.0006, *p* = 0.981, Fig. 2a), but salinity was highly significant (*df* = 1, *χ*^2^ = 12.07, *p* = < 0.001, Fig. 2a).

**Fig. 3:**
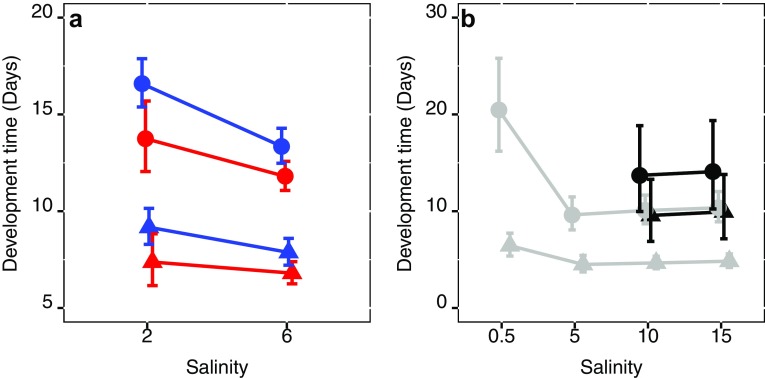
Average copepodid (triangles) and adult (circles) development time as response to salinity of **a**, the STHLM population at 15 °C (blue) and 20 °C (red). And **b,** the GOR (grey) and BB (black) population. Error bars are confidence limits

Increasing salinity and temperature shortened adult, nauplii, and copepodid development time (Table 2, Fig. 3a), and there were a tendency towards faster adult development of males than females, which was however not significant (Table 2). For adults, the effect sizes were − 2.9 and − 2.14 days for a respective salinity and temperature increase. The effect of temperature was greater than salinity for nauplii development (− 1.37 and − 1.1 days), whereas the effect of salinity was greater for adult and copepodid development (− 1.72 and − 0.85 days).

**Table 2:** General mixed models outcomes for different life-stage development time of the STHLM population estimates and CI are given in days

Reference category	Contrast	Estimate	95% CI	*z* value	*p* value
(A) Adult development					
Sal. 2, Temp. 15, sex male	(Intercept)	15.93	14.77, 17.18	71.75	< 0.0001
	Salinity 6	− 2.90	12.11, 14.01	− 4.74	< 0.0001
	Temperature 20	− 2.14	12.54, 15.17	− 3.57	< 0.001
	Sex female	1.10	15.71, 18.47	1.74	0.082
(B) Nauplii development					
Sal. 2, Temp. 15, sex male	(Intercept)	8.88	8.02, 9.82	42.30	< 0.0001
	Salinity 6	− 1.1	7.06, 8.55	− 2.35	0.019
	Temperature 20	− 1.37	6.61, 8.53	− 3.13	0.002
	Sex female	0.40	8.32, 10.35	0.87	0.386
(C) Copepodid development					
Sal. 2, Temp. 15, sex male	(Intercept)	7.17	6.40, 8.02	34.08	< 0.0001
	Salinity 6	− 1.72	4.87, 6.09	− 4.28	< 0.0001
	Temperature 20	− 0.85	5.48, 7.29	− 2.04	0.042
	Sex female	0.45	6.74, 8.60	1.04	0.299

**Fig. 4:**
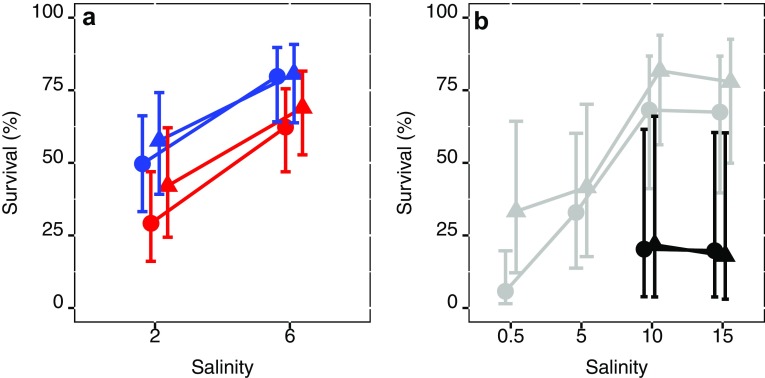
Copepodid (triangles) and adult (circles) survival in percentage as a response to salinity. For **a**, the STHLM population at 15 °C (blue) and 20 °C (red) and **b**, the GOR population in grey and the BB are in black. Error bars are confidence limits

Salinity and temperature both had an effect on adult survival, which was positively affected by an increase from 2 PSU to 6 PSU and decrease from 20 to 15 °C, a 30 and 20% increase, respectively (Table 3, Fig. 4a). Similarly, nauplii survival increased (23%) with increased salinity, but there was no effect of temperature increase (Table 3, Fig. 4a), and copepodid survival was not affected by either salinity or temperature (Table 3).

**Table 3:** Summary of regression outcomes of different life-stage survival for the different treatment combinations for the STHLM population

Reference category	Contrast	Estimate	95% CI	*z* value	*p* value
(A) Adult survival					
Temp. 15, Sal. 2	(Intercept)	0.50	0.32, 0.67	− 0.038	0.969
	Temperature 20	− 0.20	0.15, 0.47	− 2.038	0.042
	Salinity 6	0.30	0.64, 0.90	3.239	0.001
(B) Nauplii survival					
Temp. 15, Sal. 2	(Intercept)	0.58	0.39, 0.75	0.806	0.420
	Temperature 20	− 0.16	0.24, 0.63	− 1.355	0.175
	Salinity 6	0.23	0.63, 0.91	2.428	0.015
(C) Copepodid survival					
Temp. 15, Sal. 2	(Intercept)	0.90	0.81, 0.97	5.013	< 0.0001
	Temperature 20	− 0.17	0.55, 0.89	− 2.170	0.030
	Salinity 6	0.07	0.94, 0.99	2.773	0.006

Estimates and CI are given in proportions where 0 is no survivors and 1 is survival by all individuals

### Gulf of Riga and Bothnian Bay populations

We found a weak significant effect of salinity on hatching success (*df* = 3, *χ*^2^ = 7.91, *p* = 0.0479, Fig. 2b), an average increase of 0.5% PSU^−1^, and the intercept at 0.5 PSU for hatching success which was higher for the GOR population than for the BB population, 40 and 6%, respectively (*df* = 1, *χ*^2^ = 11.08, *p* < 0.001, Fig. 2b).

Adult, nauplii, and copepodid development were longer at 0.5 PSU compared to the higher salinities of 5, 10, and 15, while there was no difference between the salinities from 5 and above (Table 4, Fig. 3b), and was 10.85, 1.94, and 8.66 days shorter in 5 than in 0.5 PSU for adult, nauplii, and copepodid development, respectively.

**Table 4:** Summary of regressions parameters of development time across salinity treatments for the GOR population from general mixed model outcomes, estimates, and CI is given in days

Reference	Contrast	Estimate	95% CI	*t* value	*p* value
(A) Adult development					
Salinity 0.5	(Intercept)	20.49	16.21, 26.07	25.26	< 0.0001
	Salinity 5	− 10.85	8.05, 11.65	− 6.08	< 0.0001
	Salinity 10	− 10.44	8.54, 11.80	− 6.04	< 0.0001
	Salinity 15	− 10.12	8.83, 12.26	− 5.89	< 0.0001
Salinity 5	(Intercept)	9.63	8.05, 11.65	24.87	< 0.0001
	Salinity 10	0.42	8.54, 11.80	0.46	0.646
	Salinity 15	0.74	8.83, 12.26	0.80	0.426
Salinity 10	(Intercept)	10.06	8.54, 11.80	30.13	< 0.0001
	Salinity 15	0.32	8.83, 12.26	0.40	0.693
(B) Nauplii development					
Salinity 0.5	(Intercept)	6.44	5.34, 8.03	20.07	< 0.0001
	Salinity 5	− 1.94	3.70, 5.46	− 2.69	0.007
	Salinity 10	− 1.76	4.05, 5.40	− 2.72	0.007
	Salinity 15	− 1.64	4.12, 5.64	− 2.45	0.015
Salinity 5	(Intercept)	4.50	3.70, 5.46	15.63	< 0.0001
	Salinity 10	0.18	4.05, 5.40	0.33	0.740
	Salinity 15	0.31	4.12, 5.64	0.54	0.592
Salinity 10	(Intercept)	4.68	4.05, 5.40	21.39	< 0.0001
	Salinity 15	0.12	4.12, 5.64	0.25	0.805
(C) Copepodid development					
Salinity 0.5	(Intercept)	13.79	10.29, 18.68	17.51	< 0.0001
	Salinity 5	− 8.66	4.03, 6.57	− 6.18	< 0.0001
	Salinity 10	− 8.49	4.27, 6.49	− 6.28	< 0.0001
	Salinity 15	− 8.28	4.47, 6.85	− 6.22	< 0.0001
Salinity 5	(Intercept)	5.12	4.03, 6.57	13.56	< 0.0001
	Salinity 10	0.16	4.27, 6.49	0.25	0.801
	Salinity 15	0.38	4.47, 6.85	0.57	0.572
Salinity 10	(Intercept)	5.29	4.27, 6.49	16.72	< 0.0001
	Salinity 15	0.21	4.47, 6.85	0.36	0.716

**Fig. 5:**
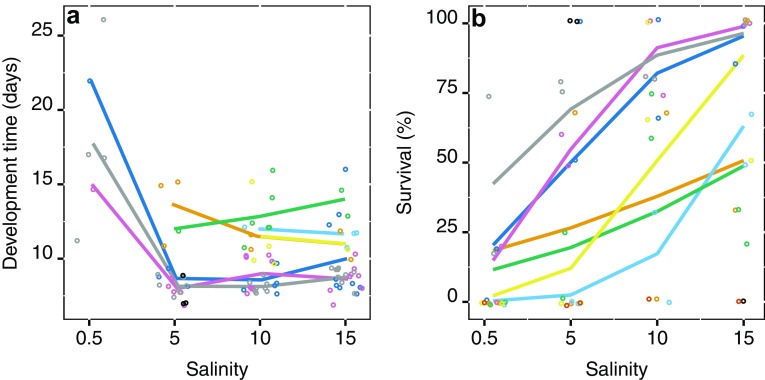
Response to salinity of the separate genotypes from the GOR population, **a** show adult development time, where points are development time per individual, and **b** show adult survival where points are average survival per vial. Color coding is the same in both panels; points show every genotype, while lines show the genotypes that have estimates across salinity

Genotype-by-salinity interaction was not significant for both adult development (*df* = 12, *χ*^2^ = 3.13, *p* = 0.514, Fig. 5a) and adult survival (*df* = 22, *χ*^2^ = 25.81, *p* = 0.259, Fig. 5b), and thus, the response to salinity was similar across genotypes, and the “best” genotype in one salinity was also best in all salinities.

**Fig. 6:**
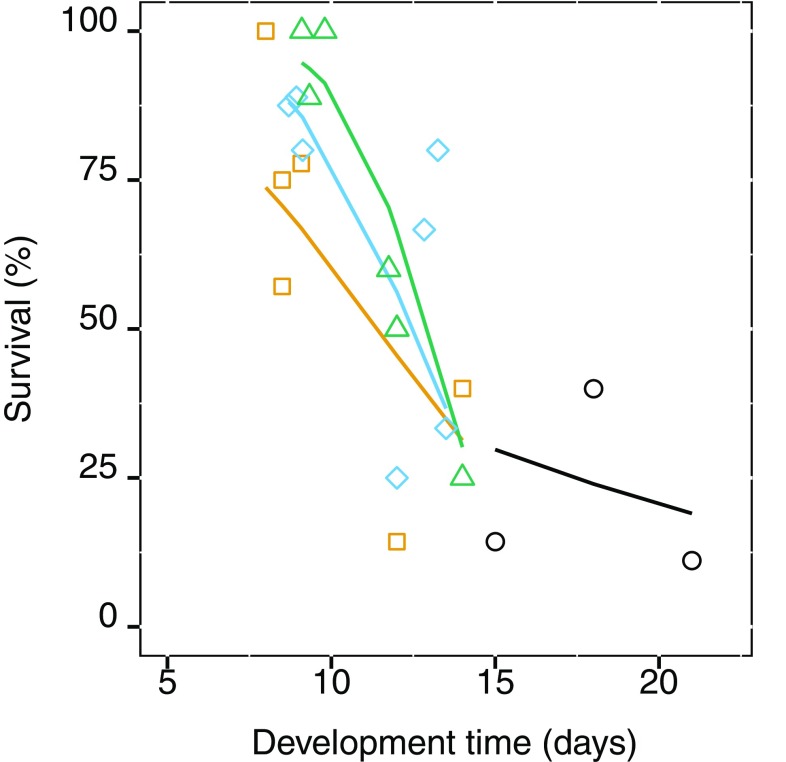
Average adult survival as a response to average adult development time in different salinities for the GOR population. Lines represent fitted values and points are observed values; the different colors are: black 0.5, orange 5, blue 10, and green 15 PSU

On genotype level, development time had a strong effect on adult survival (*df* = 1, *χ*^2^ = 17.04, *p* = < 0.0001), and there was no main effect of salinity (*df* = 1, *χ*^2^ = 0.84, *p* = 0.361), but we found a significant interaction of salinity and development time (*df* = 1, *χ*^2^ = 9.04, *p* = 0.00264). Thus, the faster the genotype develop, the more likely they are to survive, particularly in salinities from 5 PSU and above, while in lower salinity, the effect of development time decreases (Fig. 6).

Survival to adult was significantly lower in 0.5 PSU (6%) compared to the higher salinities 5 (34%), 10 (66%), and 15 PSU (67%), and lower in 5 PSU than in 10 and 15 PSU, with no difference between the two later salinities (Table 5, Fig. 4b). For nauplii, survival was significantly lower in 0.5 and 5 PSU compared to 10 and 15 PSU (Table 5), and copepodid survival differed only between 0.5 and the higher salinities 5, 10 and 15 PSU (Table 5, Fig. 4b).

**Table 5:** Summary of regression outcomes of different life-stage survival for the GOR population

Reference category	Contrast	Estimate	95% CI	*z* value	*p* value
(A) Adult survival					
Salinity 0.5	(Intercept)	0.06	0.01, 0.19	− 3.924	< 0.0001
	Salinity 5	0.28	0.12, 0.62	3.436	< 0.001
	Salinity 10	0.61	0.37, 0.88	5.410	< 0.0001
	Salinity 15	0.62	0.36, 0.88	5.369	< 0.0001
Salinity 5	(Intercept)	0.34	0.12, 0.62	− 1.178	0.239
	Salinity 10	0.34	0.37, 0.88	2.796	0.005
	Salinity 15	0.34	0.36, 0.88	2.741	0.006
Salinity 10	(Intercept)	0.67	0.37, 0.88	1.241	0.214
	Salinity 15	0.00	0.36, 0.88	0.036	0.972
(B) Nauplii survival					
Salinity 0.5	(Intercept)	0.33	0.11, 0.65	− 1.053	0.292
	Salinity 5	0.09	0.16, 0.73	0.707	0.479
	Salinity 10	0.48	0.54, 0.95	3.612	< 0.001
	Salinity 15	0.44	0.48, 0.94	3.234	0.001
Salinity 5	(Intercept)	0.42	0.16, 0.73	− 0.505	0.614
	Salinity 10	0.39	0.54, 0.95	3.257	0.001
	Salinity 15	0.35	0.48, 0.94	2.824	0.005
Salinity 10	(Intercept)	0.81	0.54, 0.95	2.261	0.024
	Salinity 15	− 0.03	0.48, 0.94	− 0.358	0.721
(C) Copepodid survival					
Salinity 0.5	(Intercept)	0.23	0.05, 0.48	− 1.681	0.093
	Salinity 5	0.63	0.63, 0.97	3.411	< 0.001
	Salinity 10	0.65	0.73, 0.96	3.681	< 0.001
	Salinity 15	0.69	0.77, 0.98	3.937	< 0.0001
Salinity 5	(Intercept)	0.86	0.63, 0.97	2.761	0.006
	Salinity 10	0.02	0.73, 0.96	0.210	0.834
	Salinity 15	0.05	0.77, 0.98	0.629	0.530
Salinity 10	(Intercept)	0.88	0.73, 0.96	3.845	< 0.001
	Salinity 15	0.03	0.77, 0.98	0.493	0.622

Estimates and CI are given in proportions where 0 is no survivors and 1 is survival by all individuals

## Discussion

Our study reveals how clutch size, hatching success, development time, and survival are affected by changing temperature, salinity, and their interactions in different populations of the calanoid copepod *E. affinis* sampled along the Baltic Sea salinity and temperature gradient. In general, our results revealed that lower salinity than ambient has detrimental effects for all population, whereas higher salinity than ambient has weak positive or no effects on life-history traits. Higher temperature decreased development time substantially and had a comparable weaker negative effect on copepod survival than salinity.

Clutch size in the STHLM population was unaffected by both salinity and temperature. Commonly, for calanoid copepods, larger females have more eggs, and females grow larger in colder temperatures (Gillooly et al. 2001; Horne et al. 2016; Mclaren 1963). The lack of clutch size differences at the temperature range used in our experiment could be due to a stationary phase at the temperature interval from 15 to 20 °C. For *E. affinis* in the Schlei Fjord (southwestern Baltic Sea), egg number and prosome size decreased with increasing temperature throughout the spring and summer (Hirche 1992). Furthermore, Hirche (1992) showed that body size and egg number follow similar fitted curves, where size and egg numbers are stationary at some temperature intervals and have inflection points at others. An opposite clutch size–temperature relation was found in an *E. affinis* population from the Seine estuary, which had larger mean clutch size at 15 °C than at 10 °C (Devreker et al. 2009). This suggests that clutch size and temperature reaction norm is population specific for *E. affinis*.

Our results showed that hatching success increase more with salinity at low temperature than at high for the STHLM population (Fig. 2a), and salinity increase had a strong positive effect on hatching success. For the GOR and BB populations, there was only a weak positive effect of salinity on hatching success, even though much lower and higher salinities than ambient were used (eight times as low and three times as high for the GOR population, and four times as low and five times as high for the BB population). Our hatching success results from Baltic Sea *E. affinis* are not in agreement with other studies of brackish water populations of *E. affinis* that found hatching success to be at large unaffected by temperature and/or salinity (Beyrend-Dur et al. 2009; Devreker et al. 2009; Diekmann et al. 2012; Lee and Petersen 2002). However, Lee et al. (2003) found a freshwater population to have lower hatching success as salinity increased. Our results suggests that lower salinity than ambient have negative effects on hatching success for *E. affinis* in the Baltic Sea. However, this likely depends on population origin and the population from the highest salinity (STHLM; Fig. 1a) showed the most negative response, but the effect is also temperature-dependent and salinity changes have larger effect at lower temperature (15 °C).

Development time is an important fitness trait in zooplankton, and intimately connected to generation time and population rate of increase, with faster development leading to higher abundances in shorter time (Allan 1976). Thus, fast developing populations will have a competitive advantage over slower developing ones, if all other possible parameters are equal. Evidence suggests that that fast development at higher temperatures tends to lead to smaller females with smaller clutch size than at lower temperatures (Mclaren 1963). However, for the calanoid copepod *Acartia hudsonica,* Avery and Dam (2007) found that the daily egg production rate increases with decreasing development time, which suggests that daily egg production will increase with increasing temperature. In zooplankton, temperature is a master factor, and increasing temperature both increases metabolism and decreases development time (Devreker et al. 2007; Gillooly et al. 2001, 2002; Vuorinen 1982). In the present study, both temperature and salinity for the STHLM population and salinity for the GOR population affected development time for all life stages. Decreasing salinity slows down development and increasing temperature speeds up development. Interestingly for the STHLM population, the magnitude of temperature and salinity effects differs between life stages. For adult and copepodid development, higher salinity decreased development time more than higher temperature. In contrast, for nauplii development, time temperature had a larger effect than salinity (Table 2, Fig. 3a). This suggests that early life stages are less affected by low salinity but more affected by high temperature compared to later life stages.

The genotype and salinity interaction is a measure of the individual genotypes’ response to salinity; with a non-significant result, we can infer a homogenous response to salinity, whereas with a significant interaction, we can infer a heterogenous response to salinity (Falconer and Mackay 1996). The interactive variation where one genotype is better in low salinity and another in high salinity implies genetic variation in the reaction norm, and a basis for selection by salinity to change the population’s mean reaction norm (Dam 2013; Lee et al. 2007). In contrast, we found no significant interaction for adult development and survival (Fig. 5a, b), and thus, there is a little variation between genotypes in their response to development and survival in different salinities. This emphasizes that the difficulty Baltic Sea *E. affinis* have with decreased salinity. Despite this, some genotypes perform better than others do, with faster development and higher survival in all salinities (Fig. 6). Selection by salinity on these better performing genotypes could, perhaps, mitigate the negative impacts of a change in salinity in a scenario of desalination in the Baltic Sea as projected by (Meier et al. 2006). The relation of salinity and development time in *E. affinis* is population specific and, therefore, likely a consequence of adaptation to local conditions (Devreker et al. 2007, 2012; Lee et al. 2003). Temperature and salinity are seemingly two master factors that control development time for *E. affinis* in the Baltic Sea.

Survival is an indicator to evaluate copepods tolerance to different environmental conditions. For the STHLM population, adult survival was about 30% higher at 6 PSU than at 2 PSU, an expected result considering that ambient salinity is about 6 PSU for this population. In comparison, a 5 °C increase from 15 to 20 °C caused 20% decrease in survival from nauplii to adult (Table 3, Fig. 4a). We observed no interactive effect of temperature and salinity for survival, even though tolerance for low and high salinities has been found to decrease as temperature increase to a stressful level in other populations (Gonzalez and Bradley 1994; Kimmel and Bradley 2001; Nagaraj 1992; Roddie et al. 1984). In the Baltic Sea, highest abundances of *E. affinis* are found at temperatures around 15 and 20 °C, e.g., in the Baltic Proper (Diekmann et al. 2012; Hernroth and Ackefors 1979). The STHLM population revealed to be sensitive to low salinity during nauplii stages but not during copepodid stages, so the effect of salinity on adult survival is largely an effect of nauplii mortality.

For the GOR population, survival was higher in ambient (5 PSU) salinity than in low salinity (0.5 PSU) and increased even further when salinity reached well above ambient conditions (10 and 15 PSU), it is not uncommon that populations perform better in conditions other than their native (Kawecki and Ebert 2004). In addition, perhaps, this is an indication of that brackish water bodies such as the Baltic Sea are marginal habitats, and that populations here live under a constant salinity compromise but also under a more relaxed competition from truly fresh or marine species that cannot tolerate these conditions. For the GOR population, survival was lower in the copepodid stages and higher in the nauplii stages (Fig. 4b) at 0.5 PSU, which is where brackish water transcends into freshwater (Remane and Schlieper 1972). Lee and Petersen (2002) found a similar pattern with lower copepodid survival due to low salinity for a North American population. Our results show opposing patterns for the STHLM and GOR populations. For individuals in the STHLM population, mortality happens mainly during their naupliar stage due to low salinity, whereas for individuals in the GOR population, mortality is higher during their copepodid stage. The previous studies of *E. affinis* populations from the Seine estuary and Chesapeake Bay have shown that mortality regardless of temperature and salinity treatment is highest in the late naupliar and early copepodid stages (Devreker et al. 2007, 2012).

## Conclusion

In this study, we show that Baltic Sea *E. affinis* populations are sensitive to lower salinities than those that they are exposed to in their present environment. The low salinity treatments used in this study are in the range of what can be found in some areas of the Baltic Sea and of what can be expected by the end of this century at the locations where our populations were sampled (Meier et al. 2006). Our results clearly show that in lower salinities, hatching success, development time, and survival are negatively affected. Furthermore, we found a uniform response of individual genotypes to salinity, where all had faster development and higher survival when salinity increased. Our results suggest that Baltic Sea *E. affinis* likely could persist desalination down to almost freshwater conditions, however, with a significant reduction in fitness, which may compromise their competitive ability under future changing environmental conditions.
